# Assessing the impact of open-label designs in patient-reported outcomes: investigation in oncology clinical trials

**DOI:** 10.1093/jncics/pkad002

**Published:** 2023-01-20

**Authors:** Jennifer Lord-Bessen, James Signorovitch, Min Yang, Mihaela Georgieva, Jessica Roydhouse

**Affiliations:** Bristol Myers Squibb, Lawrenceville, NJ, USA; Analysis Group, Inc, Boston, MA, USA; Analysis Group, Inc, Boston, MA, USA; Analysis Group, Inc, Boston, MA, USA; Menzies Institute for Medical Research, University of Tasmania, Hobart, TAS, Australia

## Abstract

**Background:**

Knowledge of treatment assignment may affect patient-reported outcomes (PROs), which is of concern in oncology, where open-label trials are common. This study measured the magnitude of open-label bias by comparing PROs for similar patient groups in oncology trials with different degrees of concealment.

**Methods:**

Individual patient data from ipilimumab arms of 2 melanoma and docetaxel arms of 2 non-small cell lung cancer (NSCLC) trials were adjusted for differences using propensity score weighting. Patients were aware of treatment assignment in CA184-022 and CheckMate 057 (open-label) but not in MDX010-20 and VITAL (blinded). Overall survival (OS) and mean changes from baseline to week 12 in the European Organization for Research and Treatment of Cancer Quality of Life Questionnaire-Core 30 (melanoma) and Lung Cancer Symptom Scale (NSCLC) scores were compared between open-label and blinded groups.

**Results:**

After adjustment, baseline characteristics were balanced between blinded (melanoma, n = 125; NSCLC, n = 424) and open-label (melanoma, n = 69; NSCLC, n = 205) groups. Study discontinuation and PRO completion rates at week 12 and OS were similar. There was no clear direction in differences in change scores between groups. In the melanoma trials, role functioning (mean = -5.2, 95% confidence interval [CI] = −15.4 to 5.0), global health status (mean = -1.3, 95% CI = -8.7 to 6.1), and pain (mean = 6.2 , 95% CI = −1.8 to 14.2) favored the blinded, whereas emotional functioning (mean = 2.2, 95% CI = -5.8 to 10.2) and diarrhea (mean = -8.3, 95% CI = −17.3 to 0.7) favored the open-label group. In the NSCLC trials, changes in dyspnea (mean = 5.4, 95% CI = -0.7 to 11.5) favored the blinded and changes in appetite (mean = -1.2, 95% CI = -8.1 to 5.7) favored the open-label group. None were clinically or statistically significant.

**Conclusions:**

This study adds to the growing evidence demonstrating that concerns regarding open-label bias should not prohibit the interpretation of large and meaningful treatment effects on PROs.

Patient-reported outcomes (PROs) are important to consider alongside clinical outcomes in drug trials, and their incorporation during drug development continues to be emphasized by regulatory agencies such as the US Food and Drug Administration and the European Medicines Agency (1,2). The European Medicines Agency recommends blinded designs for PRO measurement in oncology studies to avoid concerns about bias in open-label settings, however, in circumstances where blinding is not possible, it states that “PRO data should also be supported by objective measures” (1). Meanwhile, the US Food and Drug Administration rarely considers open-label trials to be adequate to support labeling claims based on PRO instruments because of concerns that patients’ knowledge of treatment assignment may lead to challenges in interpreting benefit (2).

As opposed to proximal outcomes to the physiology of disease and its treatment, such as symptoms, distal concepts such as emotional and social function, and quality of life (QoL) are hypothesized to be more susceptible to open-label bias, because knowledge of treatment assignment may instill hope in patients in the experimental group and disappointment in those in the control group (3). Additionally, there are concerns that in open-label settings, patients assigned to the control group may be less likely to complete PRO assessments compared with patients in the experimental group (3).

Although these concerns are prudent a priori, they warrant careful empirical evaluation to assess the actual risk, direction, and magnitude of open-label bias or risk ignoring otherwise valuable PRO information. Furthermore, given different findings regarding the effect of blinding on effect size across various clinical areas (4,5), more research is needed within specific clinical contexts, especially oncology, where unblinded trials are common (6). Prior articles and systematic reviews that have explored these concerns in cancer trials using various approaches have not found clear evidence of differential effect by blinding status (7-12). In particular, compared with blinded trials, open-label trials rarely impacted PRO completion rates (3,10) and baseline scores (10). However, a limitation of this prior work is that most of it (8-11) is based on published aggregate results, with few studies (12) using individual patient data (IPD). The focus on aggregate data, although helpful, can be limited in the ability to directly compare similar treatments with different levels of blinding while adjusting for cross-trial differences in populations. Despite this, there is a paucity of research using IPD.

Therefore, this study sought to measure the presence and magnitude of open-label bias by comparing PROs using IPD from melanoma and non-small cell lung cancer (NSCLC) trials with different degrees of concealment for treatment.

## Methods

### Study population

A targeted literature review was conducted using ClinicalTrials.gov and Project Data Sphere (PDS), a digital open-access data-sharing library, which includes de-identified IPD from academic and industry-sponsored phase 3 oncology trials. Eligible pairs of oncology trials were required to meet the following criteria: 1) included the same treatment and dose in at least 1 of the trial arms; 2) included the same cancer site and stage; 3) used the same PRO instrument; 4) differed in the level of blinding to treatment assignment; and 5) had IPD available and accessible for the treatment arm of interest (either through Bristol-Myers Squibb or publicly available through PDS). The search identified 6 trial pairs that met the inclusion criteria and were reviewed in depth. Four trial pairs were excluded because their data were subsequently retracted from PDS or were missing for the PROs. One trial pair in metastatic melanoma [MDX010-20 (NCT00094653) (13) and CA184-022 (NCT00289640) (14)] and 1 in advanced NSCLC [VITAL (NCT00532155) (15) and CheckMate 057 (NCT01673867) (16)] were comparable and met the criteria (see Table 1). Race variable was available in VITAL and CheckMate 057. In VITAL, race categories included either Caucasian/White and Other. In CheckMate 057, race categories included Asian, Black or African American, Native Hawaiian or other Pacific Islander, White, Other. For consistent reporting, we recategorized race categories in CheckMate 057 the same way as what was available in VITAL.

**Table 1.** Trial summary and design

Characteristics	Melanoma trials		NSCLC trials	
	MDX010-20 (blinded)	CA184-022 (open-label)^a^	VITAL (blinded)	CheckMate 057 (open-label)
Indication	Previously treated unresectable stage III or IV melanoma	Previously treated unresectable stage III or IV melanoma	Locally advanced or metastatic nonsquamous NSCLC	Previously treated advanced nonsquamous NSCLC
Trial phase	Phase 3	Phase 2	Phase 3	Phase 3
Treatment arms	3 mg/kg ipilimumab (ipi) + melanoma peptide vaccine (gp100 vaccine); 3 mg/kg ipi (included in the analysis);^b^ gp100 vaccine	0.3 mg/kg ipi; 3 mg/kg ipi (included in the analysis);^b^ 10 mg/kg ipi	(ziv-)aflibercept 6 mg/kg + docetaxel 75 mg/m^2^; placebo 6 mg/kg + docetaxel; 75 mg/m² (included in the analysis)^b^	nivolumab 3 mg/kg; docetaxel 75 mg/m² (included in the analysis)^b^
Trial design	Double-blind parallel arm; 3:1:1 random assignment: 3 mg/kg ipi plus gp100 vaccine, 3 mg/kg ipi alone, or gp100 vaccine alone	Double-blind parallel arm; 1:1:1 random assignment to fixed dose of ipi: 10 mg/kg, 3 mg/kg, or 0.3 mg/kg	Double-blind parallel arm; 1:1 random assignment to aflibercept or placebo administered on top of the standard docetaxel regimen (75 mg/m²)	Open-label parallel arm; 1:1 random assignment to docetaxel (75 mg/m²) or nivolumab (3 mg/kg)
Treatment schedule and duration for the treatment arm of interest	Every 3 weeks for 4 doses (ie, weeks 1, 4, 7, and 10)	Induction: 1 single dose at weeks 1, 4, 7 and 10 → total of 4 separate doses; Maintenance: single doses every 12 weeks (eg, weeks 24, 36, 48, etc.) until progression, discontinuation, study withdrawal, or end	Placebo 6 mg/kg over 1 hour IV on day 1 every 3 weeks, immediately followed by docetaxel 75 mg/m² IV over 1 hour, on day 1 until progression, discontinuation, study withdrawal, or end	Docetaxel dosed as IV over 1 hour at 75 mg/m² every 3 weeks, starting on day 1 until progression, unacceptable toxicity, study withdrawal, or end
Number and types of prior treatment received	Previous treatment with and failure, relapse, or inability to tolerate IL-2, dacarbazine, and/or temozolomide	Treatment with and failure, relapse, or inability to tolerate at least 1 prior regimen (nonexperimental or experimental)	Treatment with 1 prior platinum-based anticancer therapy	Treatment with 1 prior platinum doublet-based chemotherapy; an additional line of a separate EGFR or ALK TKI regimen permitted in patients with known EGFR mutations or ALK translocations
PRO instrument	EORTC QLQ-C30	EORTC QLQ-C30	LCSS	LCSS
Primary outcome(s)	OS	Best overall response rate	OS	OS
Secondary outcome(s)	PFS (investigator determined) Best overall response rate Disease control rate HRQoL	PFS (investigator determined) OS Disease control rate HRQoL	PFS (investigator determined) Objective response rate HRQoL	Objective response rate DOR Time to response PFS (investigator determined) Disease-related symptom improvement
Discontinuation rates at week 12	Discontinued study: 18.8% Enrolled but discontinued treatment: 15.6% Receiving treatment: 65.6%	Discontinued study: 16.9% Enrolled but discontinued treatment: 0.0% Receiving treatment: 83.1%	Discontinued study: 10.8% Enrolled but discontinued treatment: 40.8% Receiving treatment: 48.3%	Discontinued study: 13.7% Enrolled but discontinued treatment: 28.8% Receiving treatment: 57.6%
PRO completion rate at week 12	66.4%	63.8%	49.1%	45.9%

ALK = anaplastic lymphoma kinase; DOR = duration of objective response; EGFR = epidermal growth factor receptor; EORTC QLQ-C30 = European Organization for Research and Treatment of Cancer Quality of Life Questionnaire-Core 30; HRQoL = health-related quality of life; IL-2 = interleukin-2; LCSS = Lung Cancer Symptom Scale; NSCLC = non-small cell lung cancer; OS = overall survival; PFS = progression-free survival; PRO = patient-reported outcome; TKI = tyrosine kinase inhibitor.

a Patients in CA184-022 were blinded to the treatment dose but all were certain to receive ipilimumab monotherapy, therefore the 3 mg/kg ipilimumab-alone arm of the trial was considered as open-label group when compared with the 3 mg/kg ipilimumab-alone arm of MDX010-20 (blinded).

b Indicates relevant treatment arm of interest included in the analysis.

Patients were aware of the drug they received in CA184-022 (but not the dose) and in CheckMate 057 (open-label). They were unaware of the treatment and dose assignment in MDX010-20 and VITAL (blinded). Our study used data from the ipilimumab 3 mg/kg arm from CA184-022 (experimental) and MDX010-20 (active control), the docetaxel 75 mg/m² arm (active control) from CheckMate 057, and the placebo with docetaxel 75 mg/m² arm (placebo control) from VITAL.

Patients who were randomly assigned to and received the assigned treatment (melanoma: ipilimumab 3 mg/kg; NSCLC: docetaxel 75 mg/m²) and had PROs at baseline were included in the analysis. The PROs of interest were assessed at week 12 in both sets of trials. Assessments were performed regardless of treatment discontinuation for all trials except CheckMate 057, where they were performed on treatment visits only. A sensitivity analysis was conducted excluding patients who discontinued treatment but reported PRO data at week 12 (melanoma: open-label = 44, blinded = 70; NSCLC: open-label = 94, blinded = 180).

### Study outcomes

Clinical outcomes included overall survival (OS) and progression-free survival (PFS). PROs included the European Organization for Research and Treatment of Cancer (EORTC) Quality of Life Questionnaire-Core 30 (QLQ-C30) (17) for the melanoma trials, and the Lung Cancer Symptom Scale (LCSS) (18,19) for the NSCLC trials.

We evaluated the 5 functioning scales of the EORTC QLQ-C30 (physical, social, role, cognitive, and emotional functioning), the 8 symptom scales (fatigue, nausea and/or vomiting, pain, dyspnea, insomnia, appetite loss, constipation, and diarrhea), and global health status (17). All items are scored on a scale of 0 to 100. For the functioning scales and global health status, higher scores indicate better functioning; for the symptom scales, higher scores indicate higher symptom burden.

The LCSS consists of 9 items: 6 measuring lung cancer symptoms (appetite loss, fatigue, cough, dyspnea, hemoptysis, and pain) and 3 related to symptom distress, interference with activity level, and global health-related QoL (HRQoL) (18,19). Scores for each item range from 0 to 100, with 0 representing the best possible score and 100 the worst (18,19). In addition to the item scores, following standard approaches, we computed the average symptom burden index score as the mean of the 6 symptom-specific scores, with higher scores indicating greater symptom burden.

### Statistical analysis

Continuous variables were summarized using means and 95% confidence intervals (CIs), and categorical variables were summarized using frequency counts and percentages. Propensity score weighting (20,21) was used to adjust for differences in the baseline characteristics between the open-label and blinded groups. Patient characteristics that were commonly available in each trial pair were included in the propensity score models (eg, age, sex, geographic region, disease stage, Eastern Cooperative Oncology Group performance status, baseline PRO scores). Means and frequencies of baseline characteristics before and after weighting were compared using standardized mean differences (SMDs). SMDs below the commonly accepted threshold of 0.1 indicate that the characteristics of the 2 groups are balanced (22).

OS and PFS were compared before and after weighting between the open-label and blinded groups to assess similarities in the trial populations using Kaplan-Meier analysis and log-rank test. Mean differences in scores from baseline to week 12 for all PROs were calculated as the difference between the mean change scores for the open-label and blinded group. Statistical comparisons of PROs between the paired groups were conducted using Wilcoxon rank sum tests for continuous variables and χ^2^ tests for categorical variables.

Clinical significance of the differences was assessed using 1) the SD of the baseline PRO scores and 2) the minimal clinically important difference (MCID) reported in the literature. Differences of half or more of the baseline SD were considered clinically significant (23). For the EORTC QLQ-C30 scales, absolute difference of 5-10 points is considered a small change, and 10-20 points a moderate change (24); hence, MCID was defined as a difference of 10 points. For the LCSS, the MCID was defined as an absolute difference of 10 points (16,25).

All analyses were conducted using SAS Enterprise Guide version 7.15 and R version 3.6.1. Unless otherwise indicated, a *P* value less than .05 was the threshold for statistical significance based on 2-sided tests.

## Results

### Melanoma trials: MDX010-20 and CA184-022

#### Study population and baseline characteristics

In the melanoma trials, 71 patients in the open-label and 131 patients in the blinded group received ipilimumab 3 mg/kg, and of those, 69 (97.2%) and 125 (95.4%), respectively, had PRO assessments at baseline and were included in the analysis. The PRO completion rate at week 12 was similar between the open-label and blinded groups (63.8% vs 66.4%). The study discontinuation rate at week 12 was also similar (16.9% vs 18.8%).

Before weighting, the average age was 58.5 years in the open-label and 56.5 years in the blinded group, and less than half of patients were female (33.3% and 40.8%, respectively; Table 2). Large cross-trial differences prior to weighting were seen for metastatic stage at baseline and prior immunotherapy use, but these characteristics and others were balanced after weighting, with SMDs less than 0.1 and a *P* value of .05 or higher for all comparisons (Table 2).

**Table 2.** Patient characteristics before and after weighting—melanoma trials^a^

Characteristics	MDX010-20 (blinded) n = 125	CA184-022 (open-label) n = 69	Before weighting			After weighting		
			Mean difference(95% CI)	SMD	*P*	Mean difference(95% CI)	SMD	*P*
Demographics								
Age, y	56.5 (54.0 to 58.9)	58.5 (55.7 to 61.3)	2.0 (-1.7 to 5.7)	0.16	.31	0.5 (–3.2 to 4.2)	0.04	.82
Sex								
Female	40.8%	33.3%	−7.5%	0.16	.38	−4.4%	0.09	.57
Male	59.2%	66.7%	7.5%			4.4%		
Region				0.07	.91		0.02	.99
Europe	36.0%	39.1%	3.1%			−1.0%		
North America	54.4%	52.2%	−2.2%			1.0%		
Other	9.6%	8.7%	−0.9%			−0.1%		
Disease-related characteristics								
Baseline LDH				0.18	.29		0.03	.86
> ULN	36.0%	44.9%	8.9%			1.4%		
≤ ULN	64.0%	55.1%	−8.9%			−1.4%		
Baseline M-stage				0.46	.01		0.03	.98
M0 or M1a	12.0%	20.3%	8.3%			0.8%		
M1b	16.8%	30.4%	13.6%			0.4%		
M1c	71.2%	49.3%	−21.9%			−1.2%		
Baseline ECOG PS > 0	45.6%	39.1%	−6.5%	0.13	.47	−3.2%	0.06	.69
Treatment history								
Prior IL-2 therapy	22.4%	23.2%	0.8%	0.02	>.99	−2.4%	0.06	.70
Prior I-O therapy	38.4%	52.2%	13.8%	0.28	.09	−2.1%	0.04	.79
QoL								
Global health status	65.7 (61.9 to 69.6)	65.8 (60.5 to 71.1)	0.1 (-6.4 to 6.6)	0.00	.98	0.0 (-6.5 to 6.5)	0.00	.99
Functional scales								
Physical functioning	76.2 (72.3 to 80.0)	81.6 (76.7 to 86.6)	5.5 (-0.8 to 11.8)	0.25	.09	1.4 (-5.1 to 7.9)	0.06	.71
Role functioning	70.9 (65.4 to 76.4)	77.5 (70.8 to 84.3)	6.6 (-2.0 to 15.2)	0.22	.15	1.6 (-7.2 to 10.4)	0.05	.75
Emotional functioning	75.5 (71.8 to 79.3)	75.4 (69.9 to 80.8)	−0.2 (-6.9 to 6.5)	0.01	.96	0.3 (-6.0 to 6.6)	0.01	.93
Cognitive functioning	85.7 (82.6 to 88.8)	87.7 (83.3 to 92.0)	2.0 (-3.3 to 7.3)	0.11	.47	1.4 (-3.5 to 6.3)	0.08	.58
Social functioning	78.4 (74.3 to 82.6)	78.0 (71.8 to 84.3)	−0.4 (-7.8 to 7.0)	0.02	.91	0.3 (-7.0 to 7.6)	0.01	.94

CI = confidence interval; ECOG = Eastern Cooperative Oncology Group; EORTC QLQ-C30 = European Organization for Research and Treatment of Cancer Quality of Life Questionnaire-Core 30; IL-2 = interleukin-2; I-O therapy = immunotherapy; LDH = lactate dehydrogenase; PS = performance status; QoL = quality of life; SMD = standardized mean difference; ULN = upper limit of normal.

a Means and 95% confidence intervals are shown for continuous characteristics; counts and percentages are shown for categorical characteristics, unless otherwise noted. For continuous characteristics, mean differences were calculated as (open label) - (blinded) and shown with 95% confidence intervals. For categorical variables, mean differences were calculated as the differences in the percentage of patients between the open-label and blinded group. P value for comparison between the open-label and blinded group based on 2-sided test.

#### Clinical outcomes

**Figure 1.**
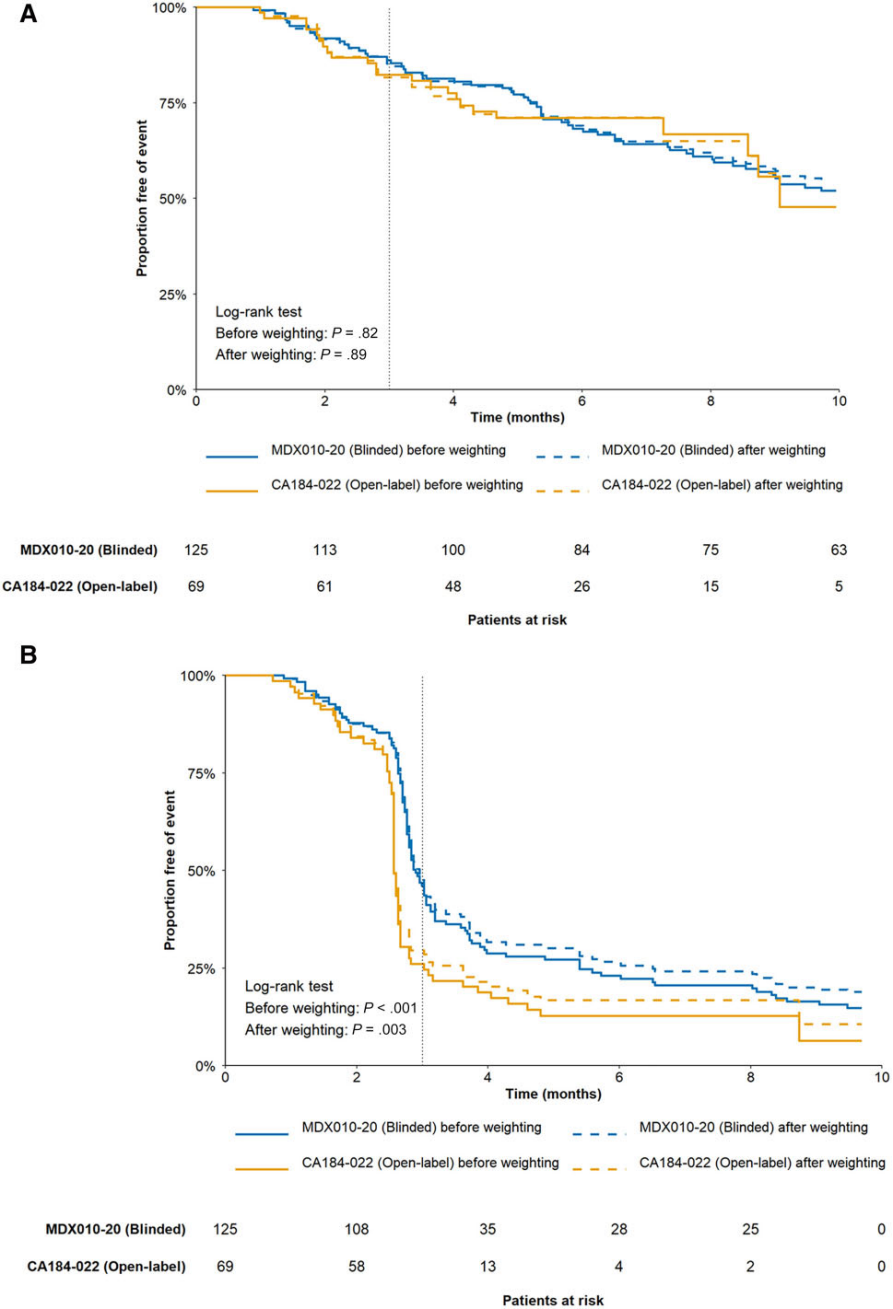
Comparison of OS and PFS in melanoma trials before and after weighting. **A)** OS of open-label and blinded groups before and after weighting. **B)** PFS of open-label and blinded groups before and after weighting. OS = overall survival; PFS = progression-free survival.

OS was similar between the open-label and blinded groups before and after weighting (after weighting: *P* = .89; Figure 1, A). However, differences in PFS remained after weighting, and PFS was lower for the open-label compared with the blinded group (*P* = .003; Figure 1, B).

#### Patient-reported outcomes

**Figure 2.**
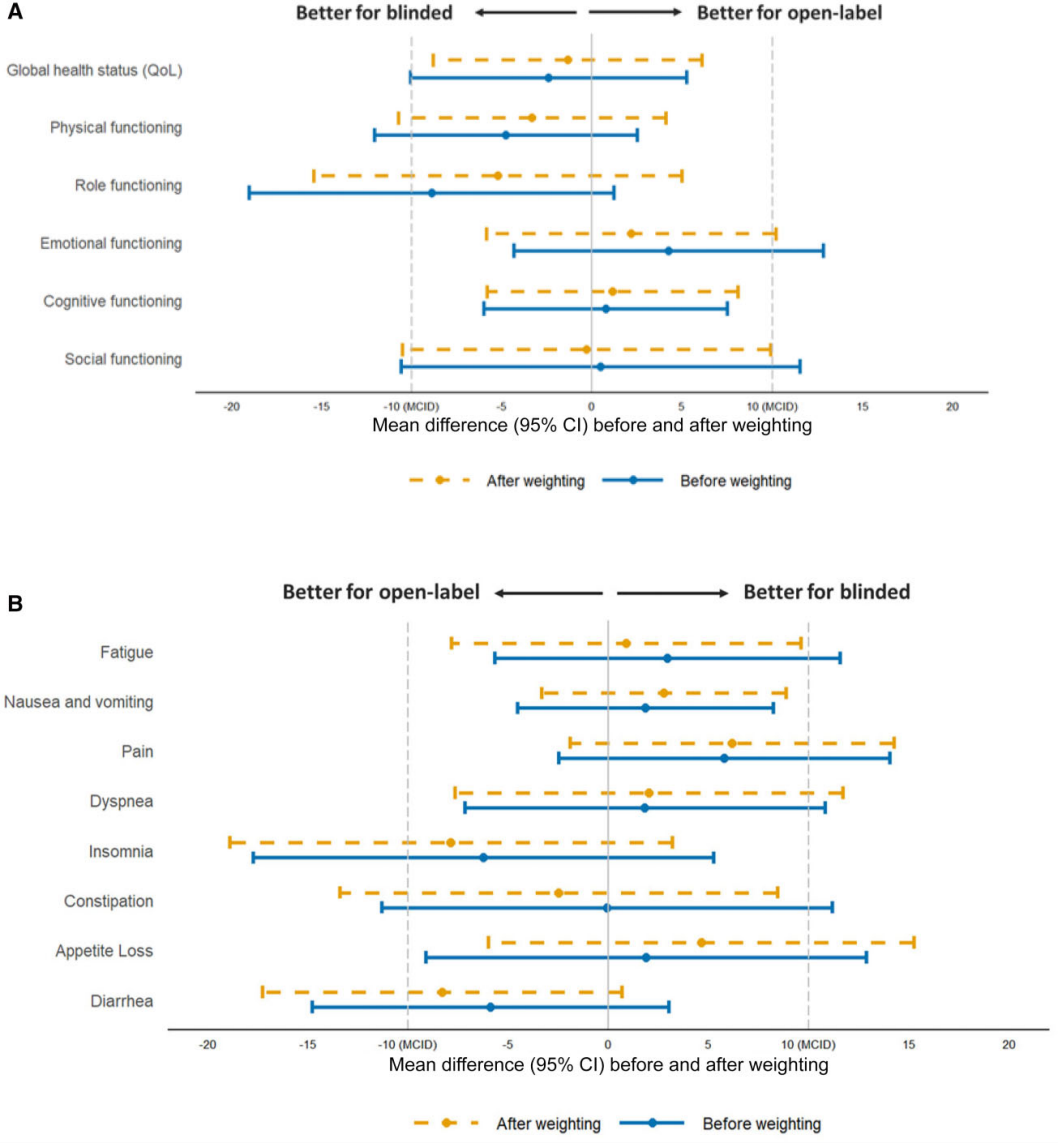
Mean differences in the EORTC QLQ-C30 from baseline to week 12 between the open-label and blinded groups. **A)** Change from baseline to week 12 in EORTC QLQ-C30 global health status and function scores in CA184-022 (open-label) vs MDX 010-20 (blinded). **B)** Change from baseline to week 12 in EORTC QLQ-C30 symptom scores in CA184-022 (open-label) vs MDX 010-20 (blinded). For the EORTC QLQ-C30 global health status and function scores, increases in score are better (ie, better functioning), whereas for symptom scores, decreases in score are better (ie, symptom reduction). CI = confidence interval; EORTC QLQ-C30 = European Organization for Research and Treatment of Cancer Quality of Life Questionnaire-Core 30; MCID = minimal clinically important difference; QoL = quality of life.

Before and after adjustment, differences in EORTC QLQ-C30 change scores between the open-label and blinded groups were not statistically significant (all *P* ≥ .05) and were all smaller than their respective half of baseline SD (Supplementary Table 1, available online). After adjustment, changes from baseline to week 12 in global health status (mean difference between groups = -1.3, 95% CI = -8.7 to 6.1), physical functioning (mean = -3.3, 95% CI = -10.7 to 4.1), role functioning (mean = -5.2, 95% CI = -15.4 to 5.0), and social functioning (mean = -0.3, 95% CI = -10.5 to 9.9) were slightly worse for the open-label group, whereas changes in emotional functioning (mean = 2.2, 95% CI = -5.8 to 10.2) and cognitive functioning (mean = 1.1, 95% CI = -6.0 to 8.2) were slightly better for the open-label than blinded group (Figure 2, A). Regarding symptom scales, changes in insomnia (mean = -7.9, 95% CI = -18.9 to 3.1), constipation (mean = -2.5, 95% CI = -13.5 to 8.5), and diarrhea (mean = -8.3, 95% CI = -17.3 to 0.7) were slightly better for the open-label group, whereas changes in fatigue (mean = 0.9, 95% CI = -7.7 to 9.5), nausea and vomiting (mean = 2.7, 95% CI = -3.4 to 8.8), pain (mean = 6.2, 95% CI = -1.8 to 14.2), dyspnea (mean = 2.0, 95% CI = -7.6 to 11.6), and appetite loss (mean = 4.6, 95% CI = -6.0 to 15.2) were slightly worse for the open-label than blinded group (Figure 2, B). All mean differences in EORTC QLQ-C30 change scores were less than 10-point MCID, with most less than 5 points. For differences greater than 5 points, role functioning and pain were better in the blinded group, and diarrhea and insomnia were better for the open-label group. Although on the individual level some patients experienced changes in their scores of more than 5 points, the proportions were similar between the 2 groups in some of the functioning (eg, global health status, emotional and cognitive functioning) and symptom domains (eg, insomnia, constipation, appetite loss; Supplementary Table 2, available online). None of the differences were statistically significant (all *P* ≥ .05) after adjustment, similar to the mean change scores.

Results from the sensitivity analysis excluding patients who reported PRO data at week 12 after having discontinued treatment were consistent with the main findings (Supplementary Figure 1, available online).

### NSCLC trials: VITAL and CheckMate 057

#### Study population and baseline characteristics

In the NSCLC trials, 268 patients in the open-label and 455 in the blinded group received 75 mg/m² docetaxel, and of those, 205 (76.5%) and 424 (93.2%), respectively, had PRO assessments at baseline and were included in the analysis. The PRO completion rate at week 12 was similar between the open-label and blinded groups (45.9% vs 49.1%). The study discontinuation rate at week 12 was also similar (13.7% vs 10.8%).

Before weighting, the average age was 62.1 years in the open-label and 59.6 years in the blinded group, and less than half of patients were female (42.9% and 34.4%, respectively; Table 3). Nearly all patients had metastatic disease (96.1% and 90.3%, respectively). Statistically significant cross-trial differences prior to weighting were seen for age, sex, region of recruitment, metastatic disease, symptom distress, and HRQoL, but these were likewise balanced following weighting, with SMDs less than 0.1 and *P* values of at least .05 for all comparisons (Table 3).

**Table 3.** Patient characteristics before and after weighting—NSCLC trials^a^

Characteristics	VITAL blinded) n = 424	CM057 (open-label) n = 205	Before weighting			After weighting		
			Mean difference^c^(95% CI)	SMD	*P*	Mean difference^c^(95% CI)	SMD	*P*
Demographics								
Age, y	59.6 (58.7 to 60.5)	62.1 (60.8 to 63.4)	2.5 (0.9 to 4.1)	0.27	.002	0.2 (-1.4 to 1.8)	0.02	.82
Sex								
Female	34.4%	42.9%	8.5%	0.18	.048	−1.0%	0.02	.83
Male	65.6%	57.1%	−8.5%			1.0%		
Race^b^				0.05	.66		0.02	.85
Non-White	11.8%	10.2%	−1.5%			−0.6%		
White	88.2%	89.8%	1.5%			0.6%		
Region				0.82	<.001		0.01	.99
Europe	73.8%	40.0%	−33.8%			0.6%		
North America	9.7%	39.5%	29.8%			−0.5%		
Other	16.5%	20.5%	4.0%			−0.1%		
Disease-related characteristics								
Metastatic disease	90.3%	96.1%	5.8%	0.23	.02	−0.3%	0.01	.93
Baseline ECOG PS > 0	68.6%	64.4%	−4.2%	0.09	.33	−0.4%	0.01	.93
LCSS								
ASBI	26.2 (24.7 to 27.8)	24.7 (22.6 to 26.9)	−1.5 (-4.2 to 1.2)	0.09	.28	0.3 (-2.2 to 2.8)	0.02	.82
Appetite loss	30.0 (27.3 to 32.6)	25.7 (22.3 to 29.2)	−4.2 (-8.5 to 0.1)	0.16	.07	−1.1 (-5.4 to 3.2)	0.04	.65
Fatigue	41.6 (39.0 to 44.2)	39.1 (35.3 to 42.8)	−2.5 (-7.0 to 2.0)	0.09	.27	−0.1 (-4.6 to 4.4)	0.00	.98
Cough	26.2 (23.7 to 28.7)	26.3 (22.6 to 30.0)	0.1 (-4.4 to 4.6)	0.00	.97	1.8 (-2.7 to 6.3)	0.07	.49
Dyspnea	30.4 (27.7 to 33.1)	29.1 (25.5 to 32.8)	−1.3 (-5.8 to 3.2)	0.05	.60	0.6 (-3.9 to 5.1)	0.02	.83
Hemoptysis	3.3 (2.3 to 4.3)	4.4 (2.7 to 6.0)	1.1 (-0.9 to 3.1)	0.09	.25	1.9 (-0.1 to 3.9)	0.16	.11
Pain	26.4 (23.7 to 29.1)	23.8 (19.9 to 27.8)	−2.6 (-7.3 to 2.1)	0.09	.29	−1.4 (-6.3 to 3.5)	0.05	.62
Symptom distress	32.2 (29.6 to 34.8)	27.2 (23.5 to 30.9)	−4.9 (-9.4 to -0.4)	0.18	.03	−0.4 (-4.9 to 4.1)	0.02	.88
Interference with activity level	37.9 (35.2 to 40.5)	36.9 (33.0 to 40.8)	−1.0 (-5.7 to 3.7)	0.03	.69	0.2 (-4.5 to 4.9)	0.01	.94
HRQoL	38.3 (35.9 to 40.8)	33.8 (30.3 to 37.2)	−4.6 (-8.9 to -0.3)	0.18	.04	−0.1 (-4.2 to 4.0)	0.00	.97

ASBI = average symptom burden index; CI = confidence interval; CM057 = CheckMate 057; ECOG = Eastern Cooperative Oncology Group; HRQoL = health-related quality of life; LCSS = Lung Cancer Symptom Scale; NSCLC = non-small cell lung cancer; PS = performance status; SMD = standardized mean difference.

a Means and 95% confidence intervals are shown for continuous characteristics; counts and percentages are shown for categorical characteristics, unless otherwise noted.

b In VITAL, race categories included either Caucasian/White and Other. In CM057, race categories included Asian, Black or African American, Native Hawaiian or other Pacific Islander, White, Other. For consistent reporting, we recategorized race categories in CM057 the same way as what was available in VITAL.

c For continuous characteristics, mean differences were calculated as (open-label) - (blinded) and shown with 95% confidence intervals. For categorical variables, mean differences were calculated as the differences in the percentage of patients between the open-label and blinded group. *P* value for comparison between the open-label and blinded group based on 2-sided test.

#### Clinical outcomes

**Figure 3.**
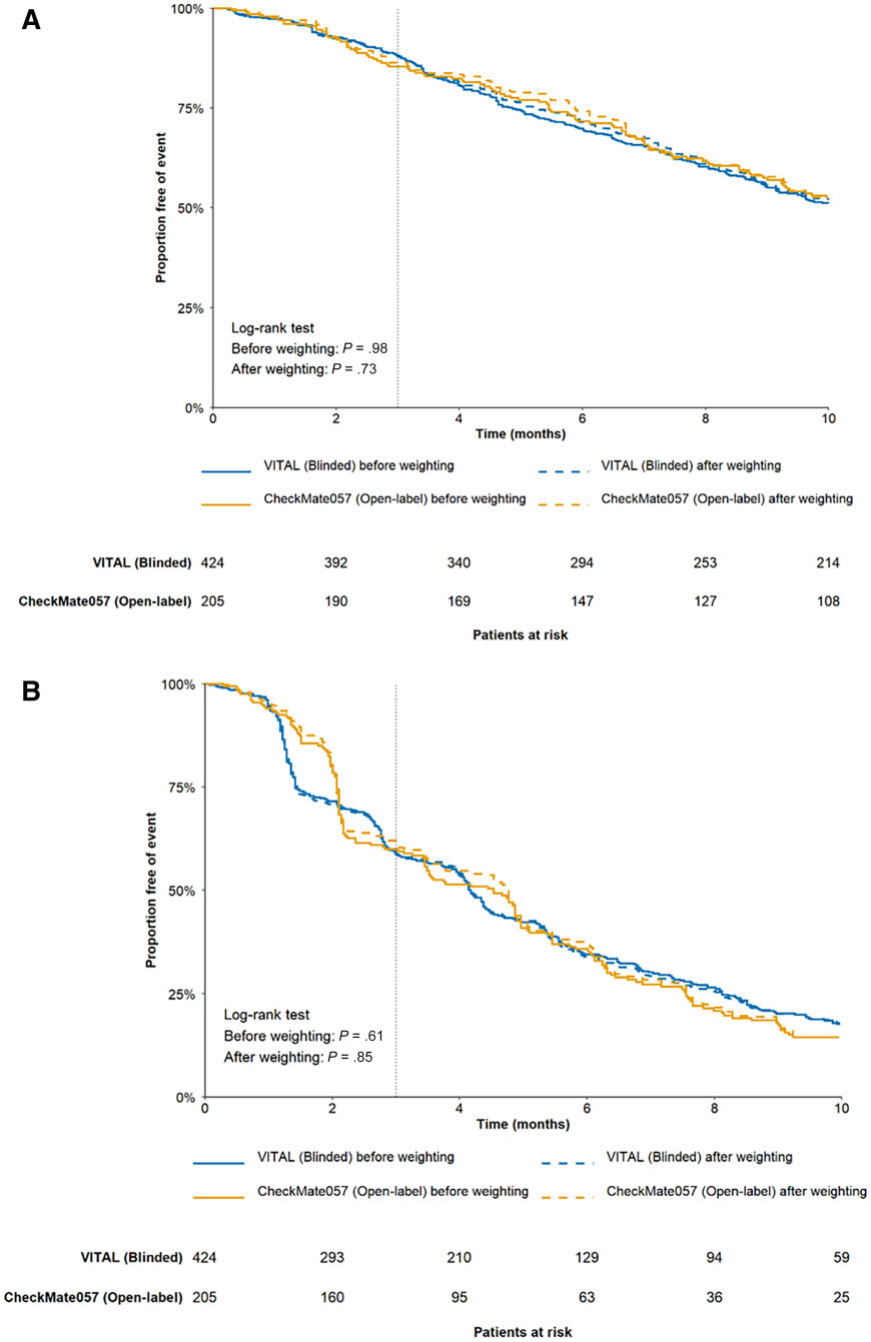
Comparison of OS and PFS in NSCLC trials before and after weighting. A) OS of open-label and blinded groups before and after weighting. **B)** PFS of open-label and blinded groups before and after weighting. NSCLC = non-small cell lung cancer; OS = overall survival; PFS = progression-free survival.

OS and PFS were similar between the open-label and blinded groups before and after weighting (after weighting: *P* = .73 for OS; *P* = .85 for PFS; Figure 3, A and B).

#### Patient-reported outcomes

**Figure 4.**
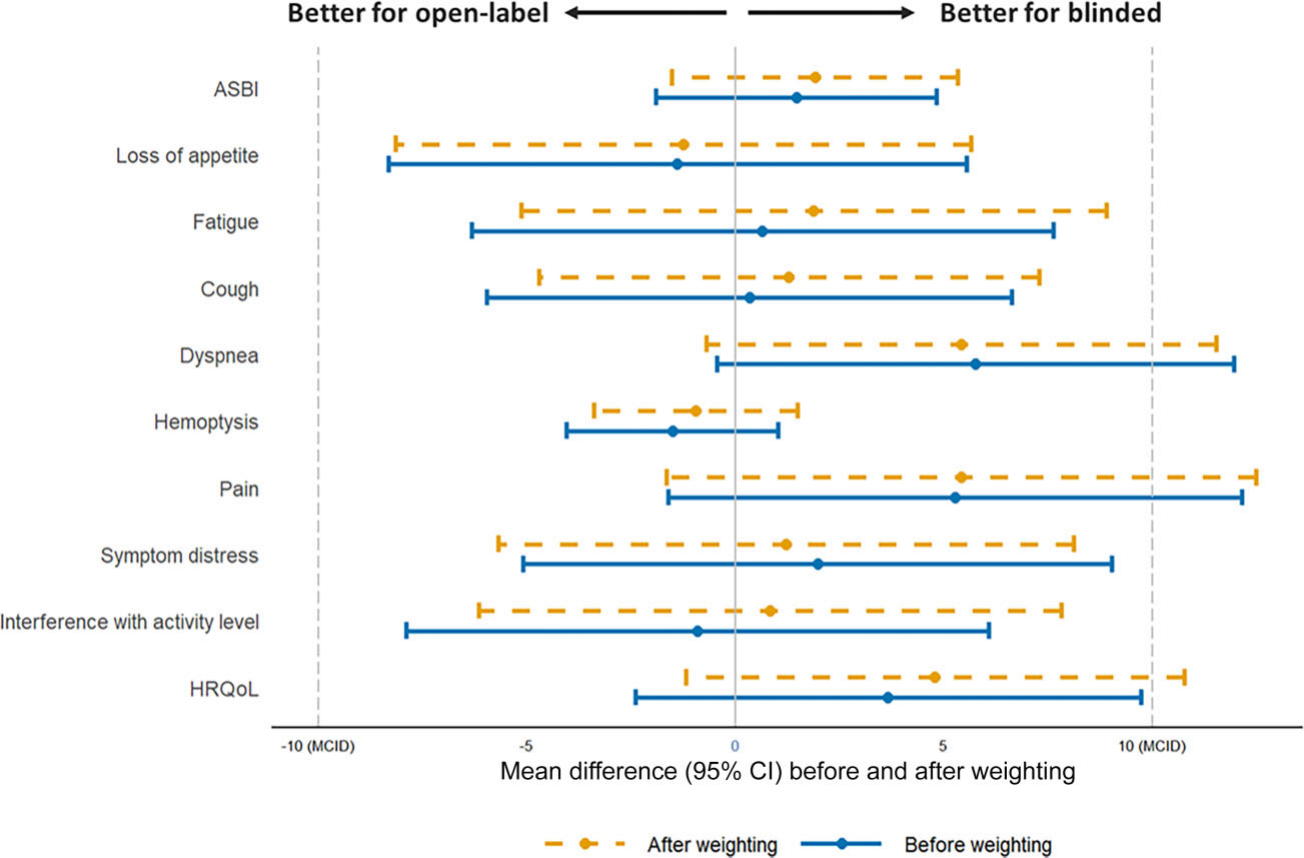
Mean differences in the LCSS from baseline to week 12 between the open-label and blinded groups. Change from baseline to week 12 in LCSS in VITAL (blinded) vs CheckMate057 (open-label). For the LCSS individual items and ASBI, decreases in scores indicate improvement. ASBI = average symptom burden index; CI = confidence interval; HRQoL = health-related quality of life; LCSS = Lung Cancer Symptom Scale; MCID = minimal clinically important difference.

Similar to the EORTC QLQ-C30 melanoma results, differences in LCSS change scores between the open-label and blinded groups were not statistically significant (all *P* ≥ .05) and were smaller than their respective half of baseline SD, both before and after adjustment (Supplementary Table 3, available online). After adjustment, changes from baseline in fatigue (mean = 1.9, 95% CI = -5.2 to 9.0), cough (mean = 1.3, 95% CI = -4.8 to 7.4), dyspnea (mean = 5.4, 95% CI = -0.7 to 11.5), pain (mean = 5.4, 95% CI = -1.7 to 12.5), symptom distress (mean = 1.2, 95% CI = -5.7 to 8.1), activity level (mean = 0.8, 95% CI = -6.3 to 7.9), HRQoL (mean = 4.8, 95% CI = -1.1 to 10.7), and average symptom burden index (mean = 1.9, 95% CI = -1.4 to 5.2) scores were slightly worse for the open-label group, and changes in appetite (mean = -1.2, 95% CI = -8.1 to 5.7) and hemoptysis (mean = -1.0, 95% CI = -3.4 to 1.4) scores were slightly better for the open-label than blinded group (Figure 4). Most mean differences in LCSS change scores were small (1-2 points), and all were less than the 10-point MCID. The largest change scores (pain and dyspnea) favored the blinded group. Although on the individual level some patients experienced changes in their scores of at least 10 points, the proportions were similar between the open-label and blinded groups for some items (eg, appetite loss, cough, hemoptysis), and none of the differences were statistically significant (*P* ≥ .05) either before or after adjustment (Supplementary Table 4, available online), similar to the mean change scores.

Results from the sensitivity analysis excluding patients who reported PRO data at week 12 after having discontinued treatment were consistent with the main findings (Supplementary Figure 2, available online).

## Discussion

In this analysis of IPD from melanoma and NSCLC trials, there was no evidence to suggest clinically or statistically significant differences between the open-label and blinded groups in any of the EORTC QLQ-C30 domains (eg, global health status, functioning, and symptom scales) and the LCSS (eg, lung cancer symptoms, symptom distress, activity level, and HRQoL). Although small, nonsignificant differences were found between the groups after adjustment, and there was no clear or consistent pattern of direction, with some differences favoring the open-label and some favoring the blinded groups. For the EORTC QLQ-C30 scales in the melanoma trials, most mean differences in change scores were less than 5 points, and those that were larger were inconsistent in direction. For the LCSS in the NSCLC trials, most mean differences between the 2 groups were small (1-2 points), and the slightly larger differences of 5 points for dyspnea and pain favored the blinded group. No differences were greater than the 10-point MCID for all trials.

The PRO findings are consistent with the lack of differential effect found with the similar OS between the open-label and blinded groups for melanoma and NSCLC and similar PFS for NSCLC, both before and after weighting. Difference in PFS was observed between the 2 groups from the melanoma trials before and after weighting. We recognize that although we made the best attempt to balance the characteristics of the patients between the trials using the propensity score weighting approach, it is possible there remain differences in baseline characteristics of the patients that were not captured and hence not available in the trial data though such variables might play an important role in PFS. To be noted, PFS was investigator determined in all 4 trials, hence, we do not expect it to be a potential source of differences (26). Excluding patients who reported PROs after discontinuing treatment in the melanoma and NSCLC trials did not change our interpretation of the PRO findings.

In this study, PRO completion rates at week 12 were similar between the open-label and blinded groups in the melanoma and NSCLC trials, which is consistent with previous findings in the literature. Though Roydhouse et al. (7) and Anota et al. (10) found some differences in PRO completion favoring the experimental arm, treatment concealment generally seemed to have little impact on PRO completion rates (7,10).

Consistent with the findings regarding PRO completion rates, the current study also found no evidence of significant bias associated with PROs because of the absence of blinding. These findings are consistent with those from Chakravarti et al. (27), who reported no clear pattern of overestimation of improvement in emotional domain scores in the open-label investigational arms compared with blinded investigational arms in 3 oncology trial pairs. In a separate analysis, Roydhouse et al. (12) used propensity score weighting and multiple imputation to compare global QoL, function, and symptoms in multiple myeloma trials using IPD and did not find evidence of consistent or meaningful differences by blinding status. Taken together, the current study adds to the growing literature demonstrating a lack of meaningful differential effect by blinding status in cancer trials, using a robust cross-trial comparison that incorporates IPD from melanoma and NSCLC trials using 2 different PRO instruments and corroboration with clinical outcomes.

Prior work has hypothesized that distal domains such as emotional function, social function, and global QoL may be more susceptible to open-label bias, with patients in the experimental arm feeling more optimistic and those in the control arm feeling disappointed with their treatment assignment (3). However, the present study found no evidence of clinically or statistically significant differences in either proximal or distal domains favoring the open-label group. Additionally, the NSCLC cross-trial comparison was based on data from the nonexperimental arms (which would have captured any disappointment patients felt regarding their treatment allocation), and there was no systematic indication of patients underreporting improvement in the open-label relative to the blinded group. Although patients in the NSCLC open-label group experienced slight improvement in some LCSS items and worsening in others, none of the differences were statistically significant.

A limitation of this study was that it compared treatment arms from separate trials. Thus, there is a risk of bias because of unobserved confounding factors even after adjusting for differences in the baseline characteristics between the trial arms, including potential biases introduced through differential missing data. As commonly seen in oncology trials, various circumstances can result in a high proportion of missing data (eg, disease progression, treatment, and study discontinuation). Although all patients with available PRO data were included in the analyses, patients who left the trial may have reported different outcomes had PRO assessments been collected. Additionally, this study was limited to trials of melanoma and NSCLC; hence, results may not be generalizable to other patient populations. Further studies of open-label bias are needed using different PRO instruments in other indications to assess the generalizability of these findings in other oncology populations.

In this evaluation of open-label bias using patient-level data, changes in EORTC QLQ-C30 domain scores in 2 melanoma trials and LCSS scores in 2 NSCLC trials were similar between patients in the open-label vs blinded groups. Any numerical differences were not consistent in direction and did not indicate clinically or statistically significant bias favoring the open-label group. This study adds to the growing body of evidence demonstrating that concerns regarding open-label bias should not prohibit the interpretation of large and meaningful treatment effects on PROs.

## Supplementary Material

pkad002_Supplementary_Data

## Data Availability

The data for the placebo plus docetaxel arm of the VITAL trial were obtained from data.projectdatasphere.org, which is maintained by *Project Data Sphere*. All other data underlying this article were provided by Bristol Myers Squibb by permission. Bristol Myers Squibb’s policy on data sharing may be found online at https://www.bms.com/researchers-and-partners/independent-research/data-sharing-request-process.html.

## Notes

**Role of the funder:** The funder did not play a role in the design of the study; the collection, analysis, and interpretation of the data; the writing of the manuscript; and the decision to submit the manuscript for publication.

**Disclosures:** JLB is an employee of Bristol Myers Squibb Company and a minor stockholder. JS, MY, and MG are employees of Analysis Group, Inc, which received consulting fees from the study sponsor to conduct this research. JR reports personal fees from Amgen, outside the submitted work, and consultancy with University of Birmingham Enterprise, outside the submitted work.

**Author contributions:** Conceptualization: JLB, JS, MY; Methodology: JLB, JS, MY, MG, JR; Formal analysis: JS, MY, MG; Data curation: JLB, JS, MY, MG, JR; Writing—original draft preparation: JS, MY, MG; Writing—review and editing: JLB, JS, MY, MG, JR.

**Acknowledgements:** This publication is based on research using information obtained from data.projectdatasphere.org, which is maintained by Project Data Sphere. Neither Project Data Sphere nor the owner(s) of any information from the website have contributed to, approved, or are in any way responsible for the contents of this publication.

Medical writing assistance was provided by Christine Tam, an employee of Analysis Group, Inc. Analytical support was provided by Anya Jiang, an employee of Analysis Group, Inc, and Ibou Dieye, who was an employee of Analysis Group, Inc, during the conduct of the study. Support for this assistance was funded by Bristol Myers Squibb.

**Prior presentations:** The analysis of the melanoma trials was presented at the 28th International Society for Quality of Life Research (ISOQOL) annual conference on October 12-28, 2021, as a virtual poster presentation.
