# Supplementary material for: Assessing the impact of open-label designs in patient-reported outcomes: investigation in oncology clinical trials
Source: JNCI Cancer Spectr. 2023 Jan 20;7(2):pkad002. doi: 10.1093/jncics/pkad002 (PMC10023242; doi:10.1093/jncics/pkad002)
Supplement: pkad002_Supplementary_Data [file pkad002_supplementary_data.pdf]

SUPPLEMENTARY TABLES AND FIGURES

Supplementary Table 1. Change in mean EORTC QLQ-C30 scores from baseline to week 12 before and after weighting

	Before Weighting				After Weighting
	Clinical Significance: Half a Baseline SD	Mean Change Score (95% CI) MDX010-20 (Blinded) N = 83	CA184-022 (Open-label) N = 44	Mean Difference[§] (95% CI)	Mean Difference[§] (95% CI)
**Quality of life**					
Global health status	11.0	-9.1 (-14.0, -4.3)	-11.6 (-17.5, -5.6)	-2.4 (-10.0, 5.2)	-1.3 (-8.7, 6.1)
**Functional scales**					
Physical functioning	10.8	-5.4 (-9.7, -1.1)	-10.2 (-16.1, -4.3)	-4.8 (-12.1, 2.5)	-3.3 (-10.7, 4.1)
Role functioning	15.2	-10.8 (-17.2, -4.4)	-19.7 (-27.5, -11.9)	-8.9 (-19.1, 1.3)	-5.2 (-15.4, 5.0)
Emotional functioning	10.9	-4.8 (-9.6, 0.0)	-0.6 (-7.7, 6.5)	4.3 (-4.3, 12.9)	2.2 (-5.8, 10.2)
Cognitive functioning	9.0	-5.7 (-9.8, -1.6)	-4.9 (-10.3, 0.4)	0.8 (-5.9, 7.5)	1.1 (-6.0, 8.2)
Social functioning	12.4	-8.1 (-14.3, -1.8)	-7.6 (-16.7, 1.5)	0.5 (-10.5, 11.5)	-0.3 (-10.5, 9.9)
**Symptom scales**					
Fatigue	11.6	12.2 (6.4, 18.0)	15.2 (8.8, 21.5)	2.9 (-5.7, 11.5)	0.9 (-7.7, 9.5)
Nausea and vomiting	9.6	4.2 (0.4, 8.1)	6.1 (1.0, 11.2)	1.8 (-4.7, 8.3)	2.7 (-3.4, 8.8)
Pain	14.0	9.0 (3.5, 14.6)	14.8 (8.7, 20.9)	5.8 (-2.4, 14.0)	6.2 (-1.8, 14.2)
Dyspnea	11.4	5.8 (-0.6, 12.2)	7.6 (1.3, 13.9)	1.8 (-7.2, 10.8)	2.0 (-7.6, 11.6)
Insomnia	14.2	10.0 (3.0, 17.1)	3.8 (-5.3, 12.9)	-6.2 (-17.8, 5.4)	-7.9 (-18.9, 3.1)
Constipation	13.3	12.5 (5.2, 19.7)	12.4 (3.8, 21.0)	-0.1 (-11.3, 11.1)	-2.5 (-13.5, 8.5)
Appetite loss	13.8	0.4 (-5.4, 6.2)	2.3 (-7.1, 11.6)	1.9 (-9.1, 12.9)	4.6 (-6.0, 15.2)
Diarrhea	9.3	8.2 (1.7, 14.6)	2.3 (-3.9, 8.4)	-5.9 (-14.7, 2.9)	-8.3 (-17.3, 0.7)

Abbreviations: CI, confidence interval; EORTC QLQ-C30, European Organization for Research and Treatment of Cancer Quality of Life Questionnaire-Core 30; SD, standard deviation.

Notes: Means and 95% CIs are shown for continuous characteristics, unless otherwise noted. [§] Mean differences were calculated as (mean change score for open-label group) – (mean change score for blinded group) and shown with 95% CIs. For global health status and the functional scales, mean differences <0 indicate that the blinded group had better outcome than the open-label group(i.e., better global health status and functioning); for the

symptom scales, mean differences >0 indicate that the blinded group had better outcome than the open-label group (i.e., symptom reduction).* p-value <0.05 for comparison between open-label and blinded group based on two-sided test.

Supplementary Table 2. Proportion of patients with small change in EORTC QLQ-C30 from baseline to week 12 before and after weighting

	Before Weighting				After Weighting	
	MDX010-20 (Blinded) N = 83	CA184-022 (Open-label) N = 44	Mean Difference	P-value	Mean Difference	P-value
**QoL**						
Global health status				0.39		0.58
>5 point decrease	55.42%	52.27%	-3.15%		-4.54%	
≤5 point change	21.69%	31.82%	10.13%		9.17%	
>5 point increase	22.89%	15.91%	-6.98%		-4.63%	
**Functional scales**						
Physical functioning				0.28		0.42
>5 point decrease	42.17%	56.82%	14.65%		13.49%	
≤5 point change	34.94%	27.27%	-7.67%		-9.40%	
>5 point increase	22.89%	15.91%	-6.98%		-4.09%	
Role functioning				0.27		0.51
>5 point decrease	42.17%	54.55%	12.38%		6.61%	
≤5 point change	39.76%	36.36%	-3.40%		0.89%	
>5 point increase	18.07%	9.09%	-8.98%		-7.50%	
Emotional functioning				0.78		0.98
>5 point decrease	36.14%	36.36%	0.22%		1.89%	
≤5 point change	34.94%	29.55%	-5.39%		-0.24%	
>5 point increase	28.92%	34.09%	5.18%		-1.64%	
Cognitive functioning				0.98		0.91
>5 point decrease	28.92%	27.27%	-1.64%		3.90%	
≤5 point change	57.83%	59.09%	1.26%		-3.54%	
>5 point increase	13.25%	13.64%	0.38%		-0.36%	
Social functioning				0.18		0.10
>5 point decrease	37.35%	31.82%	-5.53%		-5.63%	
≤5 point change	38.55%	54.55%	15.99%		19.02%	
>5 point increase	24.10%	13.64%	-10.46%		-13.39%	
**Symptom scales**						
Fatigue				0.48		0.47
>5 point decrease	21.95%	13.64%	-8.31%		-9.30%	
≤5 point change	25.61%	25.00%	-0.61%		4.83%	
>5 point increase	52.44%	61.36%	8.92%		4.47%	
Nausea and vomiting				0.49		0.24
>5 point decrease	14.46%	9.09%	-5.37%		-7.71%	
≤5 point change	59.04%	54.55%	-4.49%		-5.60%	
>5 point increase	26.51%	36.36%	9.86%		13.31%	

	Before Weighting				After Weighting	
	**MDX010-20 (Blinded) N = 83**	**CA184-022 (Open-label) N = 44**	**Mean Difference**	**P-value**	**Mean Difference**	**P-value**
Pain				<0.05		0.10
>5 point decrease	20.48%	4.55%	-15.94%		-14.62%	
≤5 point change	39.76%	47.73%	7.97%		5.61%	
>5 point increase	39.76%	47.73%	7.97%		9.01%	
Dyspnea				0.37		0.35
>5 point decrease	16.05%	6.82%	-9.23%		-9.31%	
≤5 point change	60.49%	68.18%	7.69%		5.81%	
>5 point increase	23.46%	25.00%	1.54%		3.50%	
Insomnia				0.89		0.49
>5 point decrease	15.66%	15.91%	0.25%		-2.12%	
≤5 point change	53.01%	56.82%	3.81%		11.59%	
>5 point increase	31.33%	27.27%	-4.05%		-9.47%	
Constipation				0.94		0.86
>5 point decrease	13.25%	11.63%	-1.63%		-1.49%	
≤5 point change	50.60%	53.49%	2.89%		5.56%	
>5 point increase	36.14%	34.88%	-1.26%		-4.07%	
Appetite loss				0.86		0.50
>5 point decrease	14.46%	11.36%	-3.09%		-6.95%	
≤5 point change	67.47%	68.18%	0.71%		2.25%	
>5 point increase	18.07%	20.45%	2.38%		4.70%	
Diarrhea				0.77		0.51
>5 point decrease	10.98%	13.64%	2.66%		4.14%	
≤5 point change	65.85%	68.18%	2.33%		4.62%	
>5 point increase	23.17%	18.18%	-4.99%		-8.76%	

Abbreviations: EORTC QLQ-C30, European Organization for Research and Treatment of Cancer Quality of Life Questionnaire-Core 30; MCID, minimal clinically important difference; QoL, quality of life.

Notes: Mean differences were calculated as the differences in the percentage of patients between the open-label and blinded group. P-value for comparison between open-label and blinded group based on two-sided test.

Supplementary Table 3. Change in mean LCSS scores from baseline to week 12 before and after weighting

	Before Weighting				After Weighting
		Mean Change Score (95% CI)			
	Clinical Significance: Half a Baseline SD	VITAL (Blinded) N = 208	CM057 (Open-label) N = 94	Mean Difference[§] Score (95% CI)	Mean Difference[§] Score (95% CI)
ASBI	8.1	0.4 (-1.4, 2.3)	1.9 (-0.9, 4.7)	1.5 (-1.8, 4.8)	1.9 (-1.4, 5.2)
Appetite loss	13.6	-0.2 (-3.9, 3.5)	-1.6 (-7.4, 4.3)	-1.4 (-8.3, 5.5)	-1.2 (-8.1, 5.7)
Fatigue	13.6	4.7 (0.9, 8.5)	5.4 (-0.5, 11.2)	0.6 (-6.5, 7.7)	1.9 (-5.2, 9.0)
Cough	13.4	-0.8 (-4.4, 2.8)	-0.5 (-5.6, 4.7)	0.3 (-6.0, 6.6)	1.3 (-4.8, 7.4)
Dyspnea	14.0	2.1 (-1.9, 6.0)	7.8 (3.1, 12.6)	5.8 (-0.5, 12.1)	5.4 (-0.7, 11.5)
Hemoptysis	5.5	0.9 (-0.9, 2.7)	-0.6 (-2.4, 1.2)	-1.5 (-4.0, 1.0)	-1.0 (-3.4, 1.4)
Pain	14.3	-4.5 (-7.9, -1.0)	0.8 (-5.1, 6.7)	5.3 (-1.6, 12.2)	5.4 (-1.7, 12.5)
Symptom distress	13.7	-0.6 (-4.5, 3.3)	1.3 (-4.5, 7.2)	2.0 (-5.1, 9.1)	1.2 (-5.7, 8.1)
Interference with activity level	14.2	2.7 (-0.8, 6.3)	1.8 (-4.2, 7.8)	-0.9 (-8.0, 6.2)	0.8 (-6.3, 7.9)
HRQoL	12.8	1.9 (-1.6, 5.5)	5.6 (0.7, 10.5)	3.7 (-2.4, 9.8)	4.8 (-1.1, 10.7)

Abbreviations: ASBI, average symptom burden index; CI, confidence interval; CM057, CheckMate 057; HRQoL, health-related quality of life; LCSS, Lung Cancer Symptom Scale; SD, standard deviation.

Notes: Means and 95%CIs are shown for continuous characteristics, unless otherwise noted. [§] Mean differences were calculated as (mean change score for open-label group) – (mean change score for blinded group) and shown with 95%CIs. Mean differences >0 indicate that the blinded group had better outcome than the open-label group (i.e., better HRQoL or symptom reduction). Mean differences <0 indicate that the open-label group had better outcome than the blinded group.

Supplementary Table 4. Proportion of patients with MCID in LCSS from baseline to week 12 before and after weighting

	Before Weighting				After Weighting	
	**VITAL (Blinded) N = 208**	**CheckMate 057 (Open-label) N = 94**	**Mean Difference**	**P-value**	**Mean Difference**	**P-value**
ASBI				0.54		0.23
>10 point decrease	18.81%	17.02%	-1.79%		0.49%	
≤10 point change	59.41%	55.32%	-4.09%		-10.39%	
>10 point increase	21.78%	27.66%	5.88%		9.90%	
Appetite loss				0.36		0.58
>10 point decrease	27.88%	28.72%	0.84%		1.13%	
≤10 point change	43.27%	50.00%	6.73%		5.16%	
>10 point increase	28.85%	21.28%	-7.57%		-6.29%	
Fatigue				0.40		0.38
>10 point decrease	28.50%	23.40%	-5.10%		-7.94%	
≤10 point change	35.75%	32.98%	-2.77%		0.64%	
>10 point increase	35.75%	43.62%	7.87%		7.30%	
Cough				0.80		0.91
>10 point decrease	27.05%	28.72%	1.67%		0.41%	
≤10 point change	48.79%	44.68%	-4.11%		-2.71%	
>10 point increase	24.15%	26.60%	2.44%		2.30%	
Dyspnea				0.09		0.10
>10 point decrease	28.29%	19.15%	-9.14%		-9.10%	
≤10 point change	40.98%	38.30%	-2.68%		-4.52%	
>10 point increase	30.73%	42.55%	11.82%		13.62%	
Hemoptysis				0.35		n/a[§]
>10 point decrease	2.91%	5.32%	2.41%		1.78%	
≤10 point change	92.23%	92.55%	0.32%		-0.62%	
>10 point increase	4.85%	2.13%	-2.73%		-1.16%	
Pain				0.12		0.09
>10 point decrease	26.09%	21.28%	-4.81%		-2.14%	
≤10 point change	55.56%	50.00%	-5.56%		-10.62%	
>10 point increase	18.36%	28.72%	10.37%		12.75%	
Symptom distress				0.55		0.68
>10 point decrease	27.54%	22.34%	-5.20%		-5.15%	
≤10 point change	44.44%	44.68%	0.24%		2.34%	
>10 point increase	28.02%	32.98%	4.96%		2.81%	
Interference with activity level				0.10		0.34
>10 point decrease	22.82%	29.79%	6.97%		2.50%	
≤10 point change	40.29%	27.66%	-12.63%		-10.03%	
>10 point increase	36.89%	42.55%	5.66%		7.53%	

	Before Weighting				After Weighting	
	**VITAL (Blinded) N = 208**	**CheckMate 057 (Open-label) N = 94**	**Mean Difference**	**P-value**	**Mean Difference**	**P-value**
HRQoL				0.32		0.21
>10 point decrease	26.70%	22.34%	-4.36%		-4.91%	
≤10 point change	40.78%	36.17%	-4.61%		-7.10%	
>10 point increase	32.52%	41.49%	8.97%		12.02%	

Abbreviations: ASBI, average symptom burden index; HRQoL, health-related quality of life; LCSS, Lung Cancer Symptom Scale; MCID, minimal clinically important difference.

Notes: Mean differences were calculated as the differences in the percentage of patients between the open-label and blinded group. P-value <0.05 for comparison between open-label and blinded group based on two-sided test. [§] P-values were not reported for counts <5.

Supplementary Figure 1. Mean differences in the EORTC QLQ-C30 from baseline to week 12 between the open-label and blinded groups excluding patients who discontinued treatment

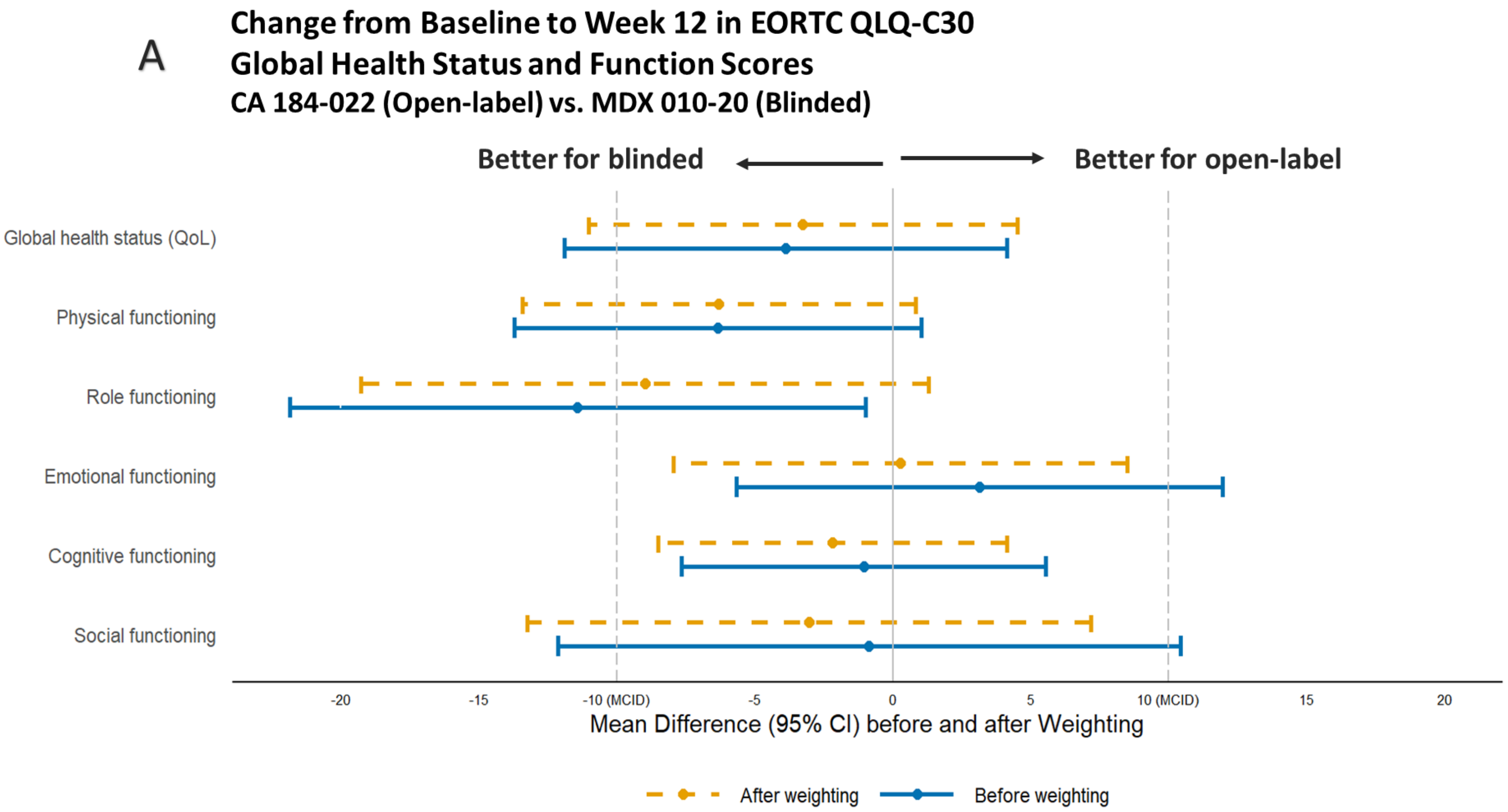

B

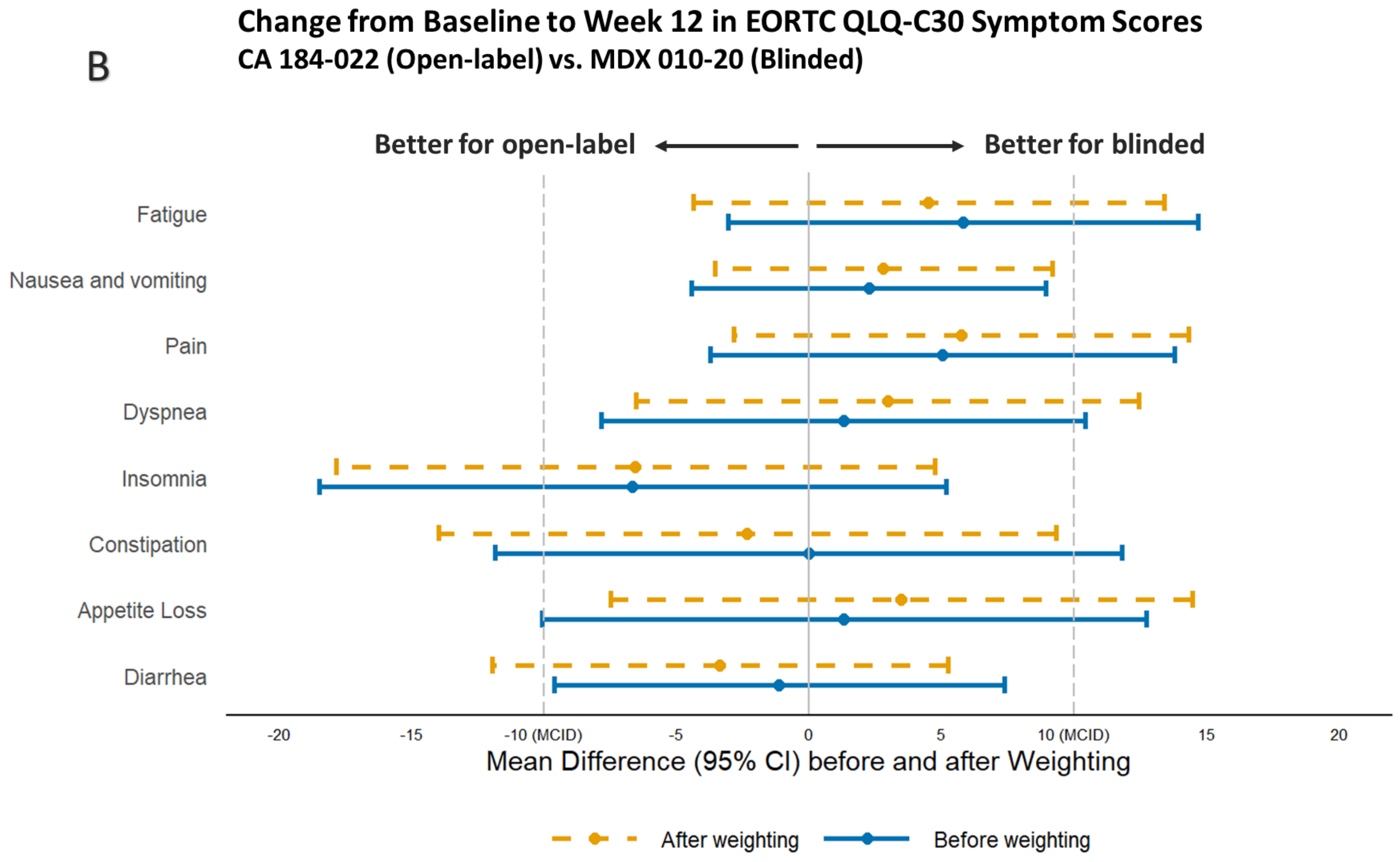

Abbreviations: CI, confidence interval; EORTC QLQ-C30, European Organization for Research and Treatment of Cancer Quality of Life Questionnaire-Core 30; MCID, minimal clinically important difference; QoL, quality of life.

Notes: 44 patients in the open-label group and 70 patients in the blinded group were included in this sensitivity analysis, with 13 patients in the blinded group being excluded as they reported PRO data at week 12 after having discontinued treatment. For the EORTC QLQ-C30 global health status and function scores increases in score are better (i.e., better functioning), while for symptom scores decreases in score are better (i.e., symptom reduction).

Supplementary Figure 2. Mean differences in the LCSS from baseline to week 12 between the open-label and blinded groups excluding patients who discontinued treatment

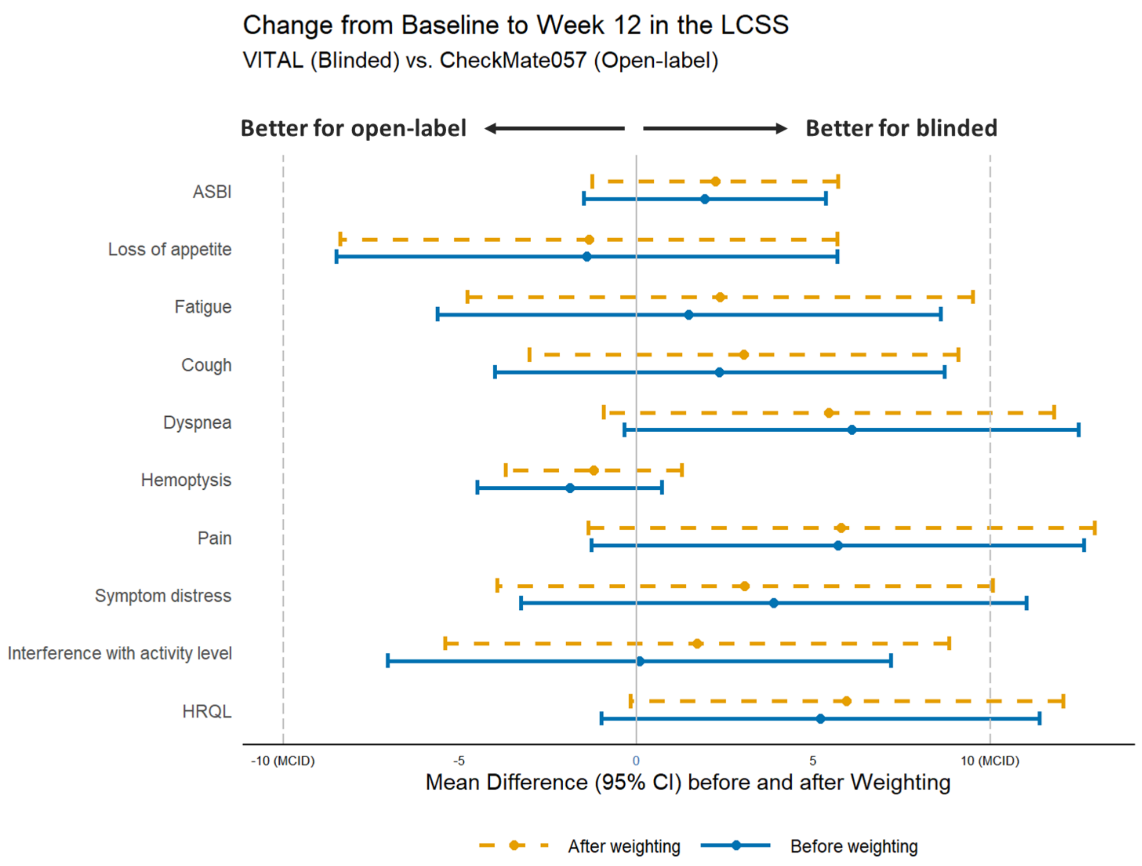

Abbreviations: CI, confidence interval; LCSS, Lung Cancer Symptom Scale; MCID, minimal clinically important difference.

Notes: 94 patients in the open-label group and 180 patients in the blinded group were included in this sensitivity analysis, with 28 patients in the blinded group being excluded as they reported PRO data at week 12 after having discontinued treatment. For the LCSS individual items and ASBI decreases in scores indicate improvement.
